# Iron levels, genes involved in iron metabolism and antioxidative processes and lung cancer incidence

**DOI:** 10.1371/journal.pone.0208610

**Published:** 2019-01-14

**Authors:** Grzegorz Mariusz Sukiennicki, Wojciech Marciniak, Magdalena Muszyńska, Piotr Baszuk, Satish Gupta, Katarzyna Białkowska, Katarzyna Jaworska-Bieniek, Katarzyna Durda, Marcin Lener, Sandra Pietrzak, Tomasz Gromowski, Karolina Prajzendanc, Alicja Łukomska, Piotr Waloszczyk, Janusz Zenon Wójcik, Rodney Scott, Jan Lubiński, Anna Jakubowska

**Affiliations:** 1 Department of Genetics and Pathology, International Hereditary Cancer Center, Pomeranian Medical University, Szczecin, Poland; 2 Read- Gene S.A., Grzepnica, Dobra Szczecińska, Poland; 3 Strand Life Sciences, Bangalore, India; 4 Independent Laboratory of Pathology, Zdunomed, Szczecin, Poland; 5 Department of General Thoracic Surgery and Transplantation, Pomeranian Medical University, Szczecin, Poland; 6 School of Biomedical Sciences, University of Newcastle, New South Wales, Newcastle, Australia; 7 Division of Molecular Medicine, NSW Health Pathology (Newcastle) New South Wales, Newcastle, Australia; 8 Independent Laboratory of Molecular Biology and Genetic Diagnostics, Pomeranian Medical University, Szczecin, Poland; University of South Alabama Mitchell Cancer Institute, UNITED STATES

## Abstract

**Background:**

Lung cancer is the most common adult malignancy accounting for the largest proportion of cancer related deaths. Iron (Fe) is an essential trace element and is a component of several major metabolic pathways playing an important role in many physiological processes. In this study we evaluated the association between Fe concentration in serum, iron metabolism parameters and genetic variaton in 7 genes involved in iron metabolism and anti-oxidative processes with the incidence of lung cancer in Poland.

**Materials and methods:**

The study included 200 lung cancer patients and 200 matched healthy control subjects. We analyzed serum iron concentration and iron metabolism parameters (TIBC, UIBC, serum ferritin and transferrin saturation), and genotyped seven variants in seven genes: *HFE*, *TFR1*, *HAMP*, *TF*, *SOD2*, *CAT* and *GPX1*.

**Results:**

Lung cancer patients compared to their matched controls had significantly higher mean serum iron level (p = 0.01), ferritin level (p = 0.007) and TIBC (p = 0.006). Analysis revealed that higher concentration of iron and ferritin (IVth quartile) compared to the lower concentration (Ist quartile) was associated with over 2-fold increased lung cancer incidence. We also found that higher transferrin saturation (p = 0.01) and lower TIBC (p<0.01) are associated with better survival of lung cancer patients. The analysis of polymorphisms in iron related genes did not reveal a significant difference between lung cancer patients and controls. However, rs10421768 in *HAMP* showed a borderline statistically significant correlation with lung cancer risk (OR = 2.83, p = 0.05).

**Conclusions:**

The results of this case control study indicate that higher body iron represented by higher Fe and ferritin levels may be associated with lung cancer incidence. Rs10421768 in *HAMP* may be associated with about 3-times higher lung cancer risk. Higher Fe body content may be associated with better survival of lung cancer patients.

## Introduction

Lung cancer is one of the most common cancers diagnosed in men and woman, and a leading cause of cancer-related deaths worldwide. The reported five-year survival rate remains dismal for lung cancer patients at 18% and is one of the lowest compared to other cancers. Although prognosis for early stage lung cancers exceeds 50%, most are detected at advanced stages when survival is very poor and drops to about 4% [1]. Cigarette smoking is a strong risk factor for lung cancer, however approximately 10% to 15% of cases occur among never smokers [2] suggesting that other independent factors may play an important role in the etiology of this disease.

Iron (Fe) is an essential component of several major metabolic pathways. It is necessary for the supply of oxygen to all cells and is involved in oxidation–reduction reactions and cellular proliferation processes [3]. Fe is required for certain enzymes such as catalases, peroxidases and cytochromes, playing an important role in antioxidant defense [3]. Although iron is an essential element for healthy life, iron overload may be harmful causing oxidative stress, DNA damage and carcinogenesis [3].

Iron homeostasis in mammals is regulated by several proteins at the levels of intestinal absorption, active cellular transport and storage. The major proteins involved in iron control are transferrin (Tf) and transferrin receptor (TFR1) for transport and utilization, ferritin for storage and hepcidine (HAMP) for controlling and coordinating these processes [4].

It is well recognized that the formation of reactive oxygen species (ROS) causing oxidative DNA damage is a significant outcome from iron overload. Elevated levels of Fe result in increased redox cycling and the production of reactive oxygen species (ROS), such as hydroxyl radicals—highly reactive species that induce lipid peroxidation and oxidative DNA damage [3]. High tissue iron concentrations have been associated with the development and progression of several pathological conditions, including certain cancers [5]. Several case-control studies have examined the association of iron levels and/or dietary intake with lung cancer [5–13]. A few studies suggest that higher dietary iron intake may be a risk factor of lung cancer [6, 9]. However, there are also reports which claim a reverse association [10, 12]. In terms of serum iron concentration, as well as other iron parameters (eg. TIBC, UIBC, transferrin saturation, ferritin level), the results were also inconclusive [5, 7, 8, 11, 13]. The observed differences between these studies may be explained by different eating habits of people belonging to different ethnicities or genetic diversity, especially in genes encoding proteins involved in iron metabolism and oxidative stress.

To date, there is no information describing the relationship between genetic variation in genes involved in iron metabolism and anti-oxidative processes, the level of iron and/or biochemical parameters of iron metabolism and the association with lung cancer in the Polish population.

The aim of this study was to evaluate body iron levels as well as iron metabolism parameters in serum and analyse genetic variations in genes involved in iron metabolism and anti-oxidative defence to assess their association with lung cancer incidence and survival in Poland.

## Materials and methods

### Study population

Study subjects were selected from the registry of the International Hereditary Cancer Centre (IHCC), Department of Genetics and Pathology, Pomeranian Medical University, Szczecin, Poland. The lung cancer case cohort consisted of 200 consecutively collected patients with lung cancer diagnosed at the Clinical Thoracic Surgery Department in Szczecin and the IHCC between August 2009 and September 2015. Patients were not eligible if they were diagnosed with any other previous malignancy, and had to be untreated before blood sampling prior to enrolment in the study. Controls were selected from a population-based study of the approximately 2 million inhabitants of the West Pomerania region in Poland, designed to identify familial aggregations of cancer syndromes. For each diagnosed lung cancer case a single cancer-free control was selected. The controls were matched to cases (1:1) with respect to year of birth (+/−3 years), sex, total number of lung cancers, total number and location of other cancers among first-degree relatives, and smoking history (pack/years +/- 10%; status: ever (current/former), never). Control participants meeting the matching criteria were identified by review of the records of the population based study and invited for interview and a blood donation. Individuals using dietary supplements of iron were excluded. The characteristics of the study groups are presented in S1 Table.

All participants were fasted for at least six hours before blood sample collection. The study was approved by the Ethics Committee of the Pomeranian Medical University in Szczecin, Poland and all participants gave informed written consent.

### Sample preparation

Blood and serum samples from lung cancer patients were obtained at the time or very shortly after diagnosis of cancer, but prior to any treatment. 10 ml of blood from cases and controls were collected with BD Vacutainer system (Becton Dickinson, USA) using certified tubes for trace metals (Royal Blue cap). Blood was then incubated at room temperature for at least 30 min. but no longer than 2 hours to clot and then was centrifuged at 1300 x g for 12 minutes. After that, serum was transferred into cryovials and placed into freezer at -80°C. The sera were stored at -80°C until analysis. At the day of analysis sera were thawed, vortexed and centrifuged at 5000 x g for 5 minutes before iron determination.

In addition, every participant enrolled in the study donated 10ml of blood to a vacutainer tube containing 1 ml of K2E (EDTA) for DNA isolation. The peripheral blood DNA was extracted using the detergent method as previously described [14] and quantified using gel electrophoresis and measurement of the A_260_/A_280_ ratio using a Perkin Elmer spectrophotometer-instrument.

### Measurement of iron levels and iron metabolism parameters

Total serum iron was determined using ICP mass spectrometer Elan DRC-e or NexION 350D (both, PerkinElmer, USA). Before each analytical run, the instrument was tuned to achieve manufacturers’ criteria. Methane was used as a reaction gas. Calibration standards were prepared using 10 μg/ml Multi-Element Calibration Standard 3 (PerkinElmer, USA) and blank reagent containing 0.65% nitric acid (Suprapur, Merc, Germany), class I deionized water (>18 MOhm, Milipore System) and 0,002% Triton X-100 (PerkinElmer, USA). Final concentration of calibration standards was 1, 5, 10 and 50 μg/l. Correlation coefficients for the calibration curves were greater than 0.999. Serum samples for total iron determination were diluted 100-times with blank reagent. Germanium isotope (Ge74) was set as an internal standard. Accuracy and precision of the analysis was tested using certified reference standards, Clincheck Plasmonorm Serum Trace Elements Level 1 (RECIPE, Germany), measured every four samples. General precision was lower than 5% RSD. Serum samples and reference material were prepared immediately before measurement. Samples from lung cancer cases and controls were measured alternately. The mean drift was used as a correction value for the samples. If the drift in the measured reference material was larger than 5% the previous samples were re-assayed and/or the instrument was re-calibrated.

Most of the circulating Fe in the body is bound to transferrin. The amount of transferrin that is available to bind to and transport iron is reflected in the total iron binding capacity (TIBC), unsaturated iron binding capacity (UIBC), or transferrin saturation. Ferritin is an iron-containing protein and is the primary form in which iron is stored. Ferritin concentration and UIBC were measured in serum samples. Then, TIBC was obtained (TIBC = UIBC+ Fe level) and used to calculate transferrin saturation (TfS) using the formula: TfS = concentration Fe/TIBC * 100 [15]. Determination of UIBC and ferritin were performed in the diagnostic laboratory “Diagnostyka”, Szczecin, Poland. The UIBC in the serum was measured by direct colorimetric method using ferrozine solution [16]. The ferritin was determined by electrochemiluminescence immunoassay (ECLIA) [17]. Both analyses were performed using Cobas C Analyzer (Roche).

### Molecular analyses

Four polymorphisms located in 4 genes involved in iron metabolism *(HFE*, *TFR1*, *TF*, *HAMP)* and 3 polymorphisms in 3 genes playing important role in anti-oxidative processes *(SOD2*, *CAT*, *GPX1)* were selected for study. All polymorphisms were functional and the frequency of the minor allele was > 5% in the Caucasian population. The rs4880 in *SOD2*, rs1050450 in *GPX1*, rs1001179 in *CAT* have been previously analyzed in lung cancer [18]. Rs1799945 in *HFE* and rs3817672 in *TFR1* have been shown to be associated with cancer risk [19]. The SNPs rs10421768 in the *HAMP* and rs1049296 in *TF* have been shown to play a role as a modulator of iron metabolism [20–21].

The genotyping of 4 genetic variants in genes involved in iron metabolism (rs1799945 in *HFE*, rs3817672 in *TFR1*, rs10421768 in *HAMP* and rs1049296 in *TF* gene) and 2 variants in genes involved in anti-oxidative processes (rs4880 in *SOD2* and rs1001179 in *CAT*) were performed using a pre-designed Genotyping Assays x 40 (Applied Biosystems). For the analysis of rs1050450 in *GPX1*, a customized assay was designed. Each reaction included 4μl of the reaction mixture and 1μl of genomic DNA. The reaction mixture consisted of 2.5μl of Master Mix (Promega), 0.625μl of Genotyping Assay (Applied Biosystems) and 1.375μl deionized water (Promega). Samples were analyzed on 384-well plates. On each plate were included positive, negative and water-blinded controls. The Real-Time PCR reaction was performed on a LightCycler 480 Instrument (Real-Time PCR System Roche Diagnostics). using Basic Software Version 1.5 (Roche Diagnostics). Each variant defined one heterozygote and two homozygote genotypes.

### Statistical analyses

The association between different categorical levels of iron concentration, disease status and genetic variants were evaluated by estimating odds ratios (OR_uni_) with 95% confidence intervals (CI) using univariable conditional logistic regression. The mean level of iron was compared between cases and controls using U-Mann-Whitney test to determine whether or not differences in any of the proportions were statistically significant.

For the assessment of association of Fe concentration with cancer occurrence, the complete data set was divided into four quartiles based on the distribution of iron levels in the cases and controls. The quartile with lowest concentration of Fe was considered as the ‘reference’ for the calculation of odd ratios (OR).

To assess a correlation of iron level and iron metabolism parameters with lung cancer tumour stage Sperman's correlation coefficients were calculated. In addition, the Mann-Whitney test was performed to compare iron and iron parameters between the four subsets of tumour stage.

The survival of lung cancer patients in relation to iron level and iron metabolism parameters was assessed using multivariable Cox proportional hazards model adjusted for age, sex and tumor stage. The Kaplan-Meier curves were generated as a graphic presentation of results. The date of death was obtained from the Polish Vital statistics registry.

The association of each of the seven tested SNPs was analyzed by univariable conditional logistic regression. The odds ratios (ORs) with their corresponding 95% CIs for the two less frequent genotypes were generated using the common homozygote genotype as the reference group.

In addition, a multivariable conditional logistic regression analysis was performed to evaluate the association between iron level, iron metabolism parameters and each tested SNP. This analysis was performed by the introduction of iron level or tested SNPs as an interaction variable to obtain adjusted odd ratios (OR_multi._). The aim of this analysis was to distinguish the bare effect of each analyzed variable after elimination of potential confounding factors.

The calculations were performed using the statistical software package R, version 3.2.3. All statistical tests were two sided, and p-values <0.05 were considered statistically significant.

## Results

In this study the association between different categorical levels of iron and 7 genetic variants in genes involved in iron metabolism or anti-oxidative processes was analyzed using 200 matched lung cancer cases and 200 controls. The lung cancer cases and controls were well matched with respect to year of birth, age, sex, family cancer history in 1^st^ degree relatives and smoking (status and life-time exposure) (S1 Table). The comparison of mean iron level and iron metabolism parameters between lung cancer patients and matched controls revealed significant differences for serum iron and ferritin levels, and TIBC (S2 Table).

### Iron levels

The mean iron level was significantly higher in the lung cancer patients compared to controls (1399.73μg/l vs. 1194.66μg/l, p = 0.01) (S2 Table). Analysis of men and women separately revealed a similar correlation, however in females the mean Fe level was lower than in males, and the correlation was not statistically significant (S2 Table).

The analysis in quartiles revealed an association of iron levels with lung cancer incidence. The odds ratio for lung cancer cases was significantly higher (OR_uni_ 2.38, p = 0.01; OR_multi_2.24, p = 0.02) in the highest quartile (>1497μg/l) compared to the lowest (<942.90μg/l) quartile in univariable and multivariable (adjusted for seven tested SNPs) analysis (S3 Table).

### Iron parameter levels

Analysis of Fe parameters revealed significantly higher mean ferritin and TIBC in lung cancer cases than in controls (S2 Table). The correlation was similar in both men and women, however, similarly to the serum Fe levels, in women the differences were not statistically significant (S2 Table).

The analysis in quartiles showed a significant association of serum ferritin with lung cancer incidence. The odds ratio for lung cancer cases was significantly greater for ferritin in the highest (>301.27μg/l) versus lowest (<107.68μg/l) quartile in univariable and multivariable analyses (OR_uni_ = 2.65, p<0.01; OR_multi_ = 2.33, p = 0.01). A similar correlation was found for TIBC (>3587.47μg/l vs. <2758.13μg/l, OR_uni_ = 2.32, p = 0.01) in univariable analysis, but not in multivariable analysis (S4 Table).

The transferrin saturation and UIBC did not differ significantly between lung cancer patients and healthy controls and there was no statistically significant association with lung cancer incidence (S2 Table and S4 Table).

### Tumour stage analyses

Analysis of correlation of iron level and iron metabolism parameters with tumour stage among lung cancer patients revealed a significant positive relationship for ferritin and a negative correlation for transferrin saturation (S5 Table). An increased level of ferritin and decreased transferrin saturation were associated with higher tumour stage. This was significant for the comparison of ferritin levels in stage 1 vs. 3 and TfS in stage 1 vs. 2, 3 and 4 (S5 Table) disease.

### Survival analysis

The mean survival time among lung cancer patients was 30 months (95%CI 26.26–33.84) and the survival rate was 38.5% for 3 years, 15.5%% for 5 years and 1.6% for 8 years.

In the multivariable Cox regression analysis there was a general trend for an association of higher body iron content with longer survival (S6 Table). In comparison to the lowest quartile, the HRs adjusted for age-, sex- and tumor stage were decreased in higher quartiles for Fe, ferritin, TIBC and TfS (S6 Table). However, a statistically significant association was detected only in quartile II of TIBC (HR 0.51, p = 0.01) and IV quartile of TfS (HR 0.55, p = 0.04) (S6 Table). The borderline significant correlation could be observed for II quartile of ferritin and UIBC (S6 Table). The trend of longer survival with higher body iron content expressed in association with increased Fe and TfS was confirmed by Kaplan-Meier analysis (S1 Fig).

### Genetic variation and lung cancer risk

Analysis of seven genetic variants showed a borderline correlation of rs10421768 in *HAMP* with lung cancer (S7 Table). The GG genotype was associated with an almost 3-times higher lung cancer risk (OR_uni_2.83, p = 0.05), but this correlation was not statistically significant when adjusted for Fe levels in multivariable analysis (OR_multi_2.90, p = 0.06). For the other 6 tested SNP’s in *TF*, *TFR1*, *HFE*, *SOD2*, *GPX1* and *CAT* genes there was no significant association with lung cancer risk (S7 Table). An analysis of the interaction of Fe concentrations and the tested SNPs failed to reveal any significant associations.

## Discussion

In this study we analyzed serum iron concentration, iron metabolism parameters and seven variants in genes associated with iron metabolism and anti-oxidative processes in a matched group of 200 lung cancer patients and 200 healthy controls. We found that serum iron levels were significantly higher in lung cancer patients than in healthy controls and its higher concentration was associated with an increased risk of lung cancer (OR_multi_2.24, p = 0.02) (S2 Table). In addition we found significant correlation of higher ferritin and TIBC with lung cancer incidence (S1 Table and S3 Table).

An association of iron with lung cancer risk was analyzed in a several observational and case-control studies. Significant association of higher Fe intake with increased lung cancer risk was reported in 2 prospective cohort studies [6, 9] and one retrospective study [22]. However, results of three other studies suggested reverse correlation of Fe intake and lung cancer risk [10, 12]. These discrepancies between the results could be caused by different eating habits of people belonging to distinct ethnicities and differences in the dietary assessment methods used, which do not control for Fe content in the food and efficiency of its absorption. Thus, the analysis of serum Fe and/or Fe metabolism parameters seams more accurate as it measures the total Fe in the body. A few studies have examined the association of serum Fe concentration and/or Fe metabolism parameters (transferrin, ferritin, TIBC) with lung cancer risk but the results are also inconclusive. A significantly higher transferrin saturation was observed in 50 men with lung cancer compared to 3113 cancer-free controls (33.9% vs. 30.07%, p<0.05) from the NHANES I cohort of over 14,000 individuals from USA [8]. The serum Fe was slightly higher (20.7μmol/l vs. 19.0μmol/l) in lung cancer patients compared to controls, but this was not statistically significant [8]. In contrast to the results reported herein, a study of over 41,000 individuals from Finland revealed slightly lower serum iron levels in >400 men with lung cancer compared to >21,000 healthy controls (114.5μg/dl vs. 115.7μg/dl), there was also no significant difference in transferrin saturation between lung cancer patients and controls (34.8% vs. 34.5%) [5]. However, they observed a significant inverse association between TIBC and lung cancer risk (RR 0.69, p<0.001) [5]. In the cohort study of over 309,000 individuals from Taiwan including 856 lung cancer patients, no statistically significant association between serum Fe and lung cancer risk was observed [7]. Significantly lower serum iron (11μmol/l vs. 19μmol/l, p<0.05) and transferrin saturation (21% vs. 30%, p<0.01) but higher ferritin level (314μg/l vs. 150/107μg/l, p<0.01) was detected in a small retrospective study of 34 consecutive patients with small cell carcinoma of the lung and 103 healthy subjects [13]. However, in another retrospective study of 30 lung cancer patients and 20 healthy controls from Turkey, serum iron was higher in affected compared to unaffected individuals (2.178μg/dl vs 1.949μg/dl, p>0.01) [11].

In the current study the measured serum iron and ferritin levels were significantly higher in lung cancer patients compared to healthy controls (S1 Table), suggesting an association of higher body Fe content with lung cancer incidence. We found over 2-times higher incidence of lung cancer (OR_uni_2.38, p = 0.01; OR_multi_2.24, p = 0.02) associated with highest quartile of Fe level (>1497μg/l) compared to lowest (<942.90μg/l) (S2 Table). Our observation is in line with results of a cohort study from USA [8] and retrospective analysis from Turkey [11], however is in opposite to 2 prospective and one retrospective studies [5, 7, 13]. Ferritin concentration is a sensitive index of the size of body iron stores. A higher ferritin levels in lung cancer patients was reported in a few retrospective studies from Denmark [13], UK [23] and China [24]. In our analysis ferritin was also higher in lung cancer patients and was positively associated with disease incidence. We found almost 3-fold greater odds ratio (OR_uni_2.65, p<0.01; OR_multi_2.33, p = 0.01) for lung cancer patients for ferritin in the highest quartile (>301.27μg/l) compared to the lowest quartile (<107.68μg/l). We also analyzed UIBC (unsaturated iron binding capacity) and transferrin saturation, but these did not show any significant differences between lung cancer patients and controls. However, we found that TIBC, which was obtained from UIBC and serum iron, was significantly higher in lung cancer patients compared to controls (3403.82μg/l vs. 3170.85μg/l, p = 0.006). This observation is not consistent with that from the Finnish cohort study, where TIBC was significantly lower in lung cancer patients than controls (333.2 μg/dl vs. 340.3μg/dl, p<0.01), and was inversely associated with lung cancer risk [5].

There is no obvious explanation for the inconsistences observed between studies in correlating serum iron and/or iron parameters with lung cancer. It could be caused by differences in study size and design, but also the sensitivity of the methods used for serum iron and/or iron parameters determination, eg. colorimetry/spectrophotometry [5, 7, 8, 13] or atomic absorption spectrophotometry [11], which differ in selectivity and sensitivity [25]. In our study serum iron concentration was determined using ICP-MS, which is very accurate method for the measurement of trace elements in biological samples [26].

In our study we evaluated the correlation of iron parameters with tumour stage among lung cancer patients. We found a significant positive relationship of ferritin and a negative correlation of transferrin saturation with lung tumour stage (S5 Table). The observed association of increased ferritin with higher stage disease is in line with the results of other studies in which higher levels of serum ferritin in advanced stages and grades of several cancers, including lung cancer, were reported [27–29]. A negative correlation of TfS with lung tumour stage confirms an association of lower body iron content with more advancer lung cancers.

Survival analysis performed in this study revealed that higher body iron content (mainly expressed by higher iron level and transferrin saturation) detected prior to treatment may be associated with decreased risk of death among lung cancer patients (S6 Table and S1 Fig and S5 Fig). A few studies have examined the influence of iron intake and iron status on cancer mortality, but results were inconclusive yet. In NHANES I prospective cohort (comprising 14,407 subjects) a reverse association of higher transferrin saturation and serum iron with an increased risk of mortality was reported [30]. In this analysis the mean transferrin saturation and iron level were higher in men who died of cancer over the study than man who did not die of cancer (32.3% vs. 30.08, p = 0.025 and 110.9μg/dl vs. 106.2 μg/dl, p = 0.03, respectively). There was also a dose-response association of increased transferrin saturation with mortality observed [30]. This was not confirmed in another prospective study performed in Netherlands comprising 394 individuals, in which hemoglobin and transferrin saturation were not associated with mortality. However, in the Dutch study a statistically significant reverse correlation of TIBC with mortality among women was observed (HR 0.05, p< 0.001 for highest vs. lowest tertile) [31]. In a Chinese prospective study of 8,291 participants a positive association of cancer mortality with iron intake in women and hemoglobin levels in men was reported [32]. In a recently published study of 298 patients with gastric cancer a statistically significant association of lower TIBC with worse disease-free and overall survival was reported (HR 2.848, p = 0.0003 and HR 3.211, p = 0.0001, respectively) [33]. The results of our study suggest, that higher body iron levels detected in cancer patients at the time of diagnosis is associated with lower mortality, and are in opposition to the NHANES I prospective study. However, they are in line with two other studies and many other reports showing an association of anemia and iron deficiency in cancer patients with ~30–40% reduced survival (for review see [34]).

Inconsistencies in the results between studies could be explained by the influence of genetic variation in genes encoding proteins involved in iron metabolism and anti-oxidative processes. To date there are limited data on the relationship between variations in the genes involved in iron metabolism and their influence on lung cancer risk. We analyzed 4 functional polymorphisms located in 4 genes—*TF*, *TFR1*, *HAMP* and *HFE*. We found a correlation of rs10421768 in *HAMP* with lung cancer risk. Homozygous carriers of the GG genotype had an almost 3-fold greater risk of lung cancer compared to carriers of the common CC genotype (OR_uni_2.83, p = 0.05). However, this correlation failed to remain significant after adjusting for iron concentration (OR_multi_2.90, p = 0.06). This polymorphism was described previously as a modulator of iron overload [21], however has not been tested with respect to its correlation with cancer risk. To our knowledge, this is the first study which reports an association of rs10421768 in *HAMP* with lung cancer. Confirmation of this relationship needs to be undertaken in a larger cohort of lung cancer patients.

The polymorphisms in *HFE*, *TFR1* and *TF* (rs1799945, rs3817672 and rs1049296, respectively) did not appear to be correlated with lung cancer in our study. None of these functional polymorphisms were previously analyzed in lung cancer patients.

Three polymorphisms located in genes involved in anti-oxidative processes—rs4880 in *SOD2*, rs1050450 in *GPX1*, rs1001179 in *CAT* were also examined as plausible variants that could alter disease risk. The rs4880 in *SOD2* and rs1050450 in *GPX1* were previously tested in lung cancer patients, however the results were inconclusive. The results of 2 meta-analyses of rs4880 in *SOD2* suggest its association with lung cancer risk [18, 35]. However, it was recently suggested that this correlation may be ethnically specific [18]. The rs1050450 in *GPX1* has been shown to be associated with an increased lung cancer risk in one study [36], but others suggests a reverse correlation [37]. The association of rs1001179 in *CAT* with lung cancer was investigated in a study from China, but no significant association was observed [38]. In the current study, we did not find any significant correlation of rs4880 in *SOD2*, rs1050450 in *GPX1* or rs1001179 in *CAT* with lung cancer risk. The results of our analyzes suggest that the tested polymorphisms in *HFE*, *TFR1*, *TF*, *SOD2*, *GPX1* and *CAT* are not associated with lung cancer risk in the Polish population. However, we cannot exclude the possibility that the current study is too small to detect any small but significant effect. Further studies on larger populations are necessary to determine a possible interaction of these SNPs, especially in *HFE*, *TFR1*, *TF*, with iron metabolism in lung cancer.

In the current study we have found an association of lung cancer incidence with higher body iron content represented by higher Fe and ferritin. Furthermore, we detected higher TIBC in lung cancer patients and its association with increased cancer incidence what suggests that not all available iron has been bound to proteins in the blood. The biologic plausibility of iron–induced carcinogenesis relies in the pro-oxidant role of iron, which may influence carcinogenesis by forming ROS leading to DNA damage. Higher body iron stores and inappropriate iron administration may overwhelm natural body defenses and promote cancer development. We observed in our study an association of higher body iron with lower mortality in lung cancer patients, which may be related to better tolerance and response to treatment. It has been reported that anemia and low hemoglobin levels may cause hypoxia which can enhance tumour progression and tumor cell resistance to chemotherapy and radiotherapy through the development of multidrug resistance [39, 40].

An advantage of our study is the complex analysis of serum iron levels and iron metabolism parameters, as well as selected variants in genes associated with iron metabolism or anti-oxidative processes in a relatively large group of lung cancer cases and controls matched with respect to year of birth, sex, total number of lung and other cancers among first degree relatives and smoking history. It is very unlikely that the detected associations could be confounded by those factors, but it is possible that other confounding variables could introduce bias.

There are limitations of the case-control approach. The serum samples for determination of iron levels and iron metabolism parameters in cases were collected at the time of lung cancer diagnosis but before therapy, so it is possible that the iron level could be influenced by the presence of cancer. The data obtained in our study are insufficient to establish a causal relationship between iron and lung cancer risk, so we are not able to determine if detected higher body iron is a marker of lung cancer diagnosis or a contributing etiologic factor.

In conclusion, the results of this study show a significant association between higher serum iron and ferritin levels with lung cancer diagnosis. Higher body iron detected at the time of diagnosis is associated with lower mortality in lung cancer patients. If confirmed, iron measurements in serum might be considered as an indicator of lung cancer risk especially in those persons who persist in smoking and valuable prognostic factor for survival of lung cancer patients. The polymorphism rs10421768 in *HAMP* may be associated with lung cancer risk, however analyses on a larger population should be performed to confirm this correlation.

## Supporting information

S1 Table Characteristic and comparison of lung cancer patients and controls. (PDF)

S2 Table Serum iron levels and iron metabolism parameters in lung cancer patients and controls. (PDF)

S3 Table Probability of lung cancer depending on iron concentration. (PDF)

S4 Table Iron metabolism parameters in lung cancer cases and controls. (PDF)

S5 Table Correlation of stage with iron level and iron parameters. (PDF)

S6 Table The survival analysis of lung cancer cases depending on iron level and iron metabolism parameters. (PDF)

S7 Table Genotype frequency in 7 analyzed genes and lung cancer risk. (PDF)

S1 Fig Kaplan-Meier curves of 8-years survival depending on iron level. (PDF)

S2 Fig Kaplan-Meier curves of 8-years survival depending on ferritin level. (PDF)

S3 Fig Kaplan-Meier curves of 8-years survival depending on UIBC. (PDF)

S4 Fig Kaplan-Meier curves of 8-years survival depending on TIBC. (PDF)

S5 Fig Kaplan-Meier curves of 8-years survival depending on transferrin saturation. (PDF)
