# Supplementary material for: Iron levels, genes involved in iron metabolism and antioxidative processes and lung cancer incidence
Source: PLoS One. 2019 Jan 14;14(1):e0208610. doi: 10.1371/journal.pone.0208610 (PMC6331102; doi:10.1371/journal.pone.0208610)
Supplement: S1 Table — (PDF) [file pone.0208610.s001.pdf]

S1 Table. Characteristic and comparison of lung cancer patients and controls.

Characteristic	Cases, n=200	Controls, n=200	p-value[a]
Mean year of birth (range)	1948 (1928-1977)	1948 (1931-1976)	0.66
Mean age (range)	67.94 (38-87)	67.62 (39-84)	0.67
Sex, n (%)			
Male	151 (75.5)	151 (75.5)	-
Female	49 (24.5)	49 (24.5)	-
Smoking			
Current (%)	73 (36.5)	79 (39.5)	-
Former (%)	114 (57)	105 (52.5)	-
Never, n (%)	13 (6.5)	16 (8)	-
Mean pack-years (range)	30.36 (0-110)	30.06 (0-150)	0.96
Histology, n (%)			
Squamous cell carcinoma	77 (38.5)	-	-
Adenocarcinoma	80 (40)	-	-
Small cell carcinoma	7 (3.5)	-	-
Large cell carcinoma	6 (3)	-	-
Other	22 (11)	-	-
Missing	8 (4)	-	-
Tumour stage, n (%)			
I	59 (29.5)	-	-
II	38 (19)	-	-
III	68 (34)	-	-
IV	26 (13)	-	-
Missing	9 (4.5)		
Survival, n (%)			
Live	65 (32.5)	-	-
Dead	135 (67.5)	-	-
Family cancer history in 1st degree relatives, n (%)			
Lung cancer	35 (17.5)	30 (15)	-
Other cancer	40 (20)	40 (20)	-

[a]p-value obtained using U-Mann-Whitney test
