# Supplementary material for: Iron levels, genes involved in iron metabolism and antioxidative processes and lung cancer incidence
Source: PLoS One. 2019 Jan 14;14(1):e0208610. doi: 10.1371/journal.pone.0208610 (PMC6331102; doi:10.1371/journal.pone.0208610)
Supplement: S2 Table — (PDF) [file pone.0208610.s002.pdf]

S2 Table. Serum iron levels and iron metabolism parameters in lung cancer patients and controls.

Group	Cases, n=200	Controls, n=200	p-value[a]
Mean serum iron level (µg/l)			
All	1399.73	1194.66	**0.01**
Male	1436.71	1223.69	**0.003**
Female	1285.76	1105.21	0.96
Mean serum ferritin level (µg/l)			
All	260.69	217.82	**0.007**
Male	284.10	241.30	**0.007**
Female	188.56	145.47	0.38
Mean serum UIBC level (µg/l)			
All	2004.09	1976.19	0.47
Male	1921.56	1945.26	0.28
Female	2258.43	2071.49	0.90
Mean serum TIBC level (µg/l)[b]			
All	3403,82	3170,85	**0.006**
Male	3358,27	3168,95	**0.003**
Female	3544,19	3176,70	0.73
Mean serum transferrin saturation level (TfS) (%)[b]			
All	40.32	38.88	0.13
Male	41.95	40.10	0.06
Female	35.29	35.15	0.68

[a] p-value obtained using U-Mann-Whitney test

[b] values calculated from formulas: TIBC=UIBC+serum iron; TfS=serum iron/TIBC*100%
