# Supplementary material for: Iron levels, genes involved in iron metabolism and antioxidative processes and lung cancer incidence
Source: PLoS One. 2019 Jan 14;14(1):e0208610. doi: 10.1371/journal.pone.0208610 (PMC6331102; doi:10.1371/journal.pone.0208610)
Supplement: S3 Table — (PDF) [file pone.0208610.s003.pdf]

S3 Table. Probability of lung cancer depending on iron concentration.

Quartile	Iron concentration (µg/l)	Cases, n=200 (%)	Controls, n=200 (%)	OR_{uni} (95%CI)[a]	p-value	OR_{multi} (95%CI)[b]	p-value
I	<942.90	47 (23.5)	53 (26.5)	1	-	1	-
II	942.91-1203.83	43 (21.5)	57 (28.5)	0.91 (0.48 - 1.71)	0.77	0.73 (0.36-1.45)	0.37
III	1203.84-1496.57	43 (21.5)	57 (28.5)	0.92 (0.50 - 1.70)	0.80	0.76 (0.39 - 1.48)	0.42
**IV**	**>1497**	**67 (33.5)**	**33 (16.5)**	**2.38 (1.26 – 4.51)**	**0.01**	**2.24 (1.12 – 4.46)**	**0.02**

[a]OR_{uni} univariable conditional logistic regression

[b]OR_{multi} multivariable conditional logistic regression (adjusted for seven tested SNP's).
