# Supplementary material for: Iron levels, genes involved in iron metabolism and antioxidative processes and lung cancer incidence
Source: PLoS One. 2019 Jan 14;14(1):e0208610. doi: 10.1371/journal.pone.0208610 (PMC6331102; doi:10.1371/journal.pone.0208610)
Supplement: S4 Table — (PDF) [file pone.0208610.s004.pdf]

S4 Table. Iron metabolism parameters in lung cancer cases and controls.

Quartile	Concentration	Cases, n=200 (%)	Controls, n=200 (%)	OR_{uni} (95%CI)[a]	p-value	OR_{multi} (95%CI)[b]	p-value
Ferritin (µg/l)							
I	<107.68	45 (22.5)	55 (27.5)	1	-	1	-
II	107.68-185.55	45 (22.5)	55 (27.5)	1.06 (0.59 - 1.89)	0.85	1.01 (0.55-1.87)	0.77
III	185.55-301.27	45 (22.5)	55 (27.5)	1.10 (0.61 - 1.98)	0.75	1.06 (0.57 - 1.94)	0.80
**IV**	**>301.27**	**65 (32.5)**	**35 (17.5)**	**2.65 (1.39 – 5.03)**	**<0.01**	**2.33 (1.19 – 4.55)**	**0.01**
UIBC (µg/l)							
I	<1598.25	44 (22)	56 (28)	1	-	1	-
II	1598.25-1937.50	55 (27.5)	45(22.5)	1.58 (0.90 - 2.80)	0.12	1.74 (0.95 - 3.18)	0.07
III	1937.50-2249.75	50 (25)	50 (25)	1.26 (0.73 - 2.19)	0.41	1.36 (0.76 - 2.44)	0.29
IV	>2249.75	51 (25.5)	49 (24.5)	1.36 (0.76 - 2.43)	0.29	1.46 (0.79 - 2.72)	0.23
TIBC (µg/l)							
I	<2758.13	40 (20)	60 (30)	1	-	1	-
II	2758.13-3143.05	52 (26)	48 (24)	1.62 (0.93-2.80)	0.09	1.65 (0.92-2.94)	0.09
III	3143.05-3587.47	47 (23.5)	53 (26.5)	1.29 (0.76-2.20)	0.35	1.19 (0.68-2.06)	0.55
**IV**	**>3587.47**	**61 (30.5)**	**41 (20.5)**	**2.32 (1.31-4.11)**	**0.01**	1.67 (0.87-3.21)	0.13
TfS (%)							
I	<31.35	49 (24.5)	51 (25.5)	1	-	1	-
II	31.35-38.70	43 (21.5)	57 (28.5)	0.79 (0.45 - 1.40)	0.42	0.79 (0.42-1.50)	0.47
III	38.70-46.43	49 (24.5)	51 (25.5)	1.05 (0.58 - 1.90)	0.87	0.86 (0.41 – 1.82)	0.70
IV	>46.43	59 (29.5)	41 (20.5)	1.61 (0.88 – 2.96)	0.12	0.90 (0.39 – 2.07)	0.81

[a]OR_{uni} univariable conditional logistic regression

[b]OR_{multi} multivariable conditional logistic regression (adjusted for iron concentration).
