# Supplementary material for: Iron levels, genes involved in iron metabolism and antioxidative processes and lung cancer incidence
Source: PLoS One. 2019 Jan 14;14(1):e0208610. doi: 10.1371/journal.pone.0208610 (PMC6331102; doi:10.1371/journal.pone.0208610)
Supplement: S5 Table — (PDF) [file pone.0208610.s005.pdf]

S5 Table. Correlation of stage with iron level and iron parameters

Value	Iron	Ferritin	TIBC	UIBC	TfS
Correlation coefficient	-0.12	**0.18**	0.01	0.12	**-0.14**
Significance*	0.35	**0.01**	0.83	0.13	**0.02**
Stage	Significance**				
1 vs. 2	0.07	0.36	0.48	0.10	<**0.01**
1 vs. 3	0.08	<**0.01**	0.80	0.12	**0.03**
1 vs. 4	0.27	0.29	0.69	0.06	**0.04**
2 vs. 3	0.64	0.15	0.41	0.06	0.08
2 vs. 4	0.56	0.64	0.89	0.51	0.55
3 vs. 4	0.74	0.46	0.64	0.42	0.44

* Spearman correlation analysis; ** Mann-Whitney test
