# Supplementary material for: Iron levels, genes involved in iron metabolism and antioxidative processes and lung cancer incidence
Source: PLoS One. 2019 Jan 14;14(1):e0208610. doi: 10.1371/journal.pone.0208610 (PMC6331102; doi:10.1371/journal.pone.0208610)
Supplement: S6 Table — (PDF) [file pone.0208610.s006.pdf]

S6 Table. The survival analysis of lung cancer cases depending on iron level and iron metabolism parameters.

Quartile	Concentration	HR (95%CI)	p-value
Fe (μg/l)			
I	<959.92	1	-
II	960.86-1298.54	0.90 (0.54 - 1.49)	0.68
III	1305.96-1619.46	0.81 (0.48 - 1.37)	0.44
IV	>1628.62	0.60 (0.35 – 1.04)	0.07
Ferritin (μg/l)			
I	<113.5	1	-
II	114.2-212.6	1.76 (1.01 – 3.06)	0.05
III	214-334.4	1.19 (0.68 – 2.09)	0.54
IV	>334.9	1.07 (0.61 – 1.88)	0.80
UIBC (μg/l)			
I	<1628	1	-
II	1637-1929	0.57 (0.33 – 0.98)	0.05
III	1936-2248	0.74 (0.44 – 1.24)	0.25
IV	>2265	0.92 (0.54 – 1.55)	0.74
TIBC (μg/l)			
I	<2839.8	1	-
**II**	**2863.93-3176.28**	**0.51 (0.30-0.85)**	**0.01**
III	3180.84-3708.95	0.61 (0.37-1.02)	0.06
IV	>3733.42	0.63 (0.38-1.05)	0.08
TfS (%)			

I	<31.71	1	-
II	32.02-40.03	1.15 (0.67 - 1.85)	0.67
III	40.73-48.2	1.05 (0.62 - 1.76)	0.86
**IV**	**>48.67**	**0.55 (0.31 – 0.97)**	**0.04**
