# Supplementary material for: Iron levels, genes involved in iron metabolism and antioxidative processes and lung cancer incidence
Source: PLoS One. 2019 Jan 14;14(1):e0208610. doi: 10.1371/journal.pone.0208610 (PMC6331102; doi:10.1371/journal.pone.0208610)
Supplement: S7 Table — (PDF) [file pone.0208610.s007.pdf]

S7 Table. Genotype frequency in 7 analyzed genes and lung cancer risk

Genotypes	Cases, n=200 (%)	Controls, n=200 (%)	OR_{uni}(95%CI)[a]	p-value	OR_{multi} (95%CI)[b]	p-value
rs1049296 in *TF*						
CC	138(69)	138(69)	1	-	1	-
CT	55(27.5)	56(28)	0.98 (0.63-1.54)	0.94	0.88 (0.55 - 1.42)	0.61
TT	7(3.5)	6(3)	1.19 (0.36-3.95)	0.77	1.15 (0.34 - 3.95)	0.82
rs3817672 in *TFR1*						
TT	53(26.5)	64(32)	1	-	1	-
TC	102(51)	106(53)	1.14 (0.74- 1.75)	0.56	1.08 (0.69 - 1.69)	0.75
CC	45(22.5)	30(15)	1.76 (0.99- 3.13)	0.06	1.74 (0.96 - 3.18)	0.07
rs1799945 in *HFE*						
CC	152(76)	148(74)	1	-	1	-
CG	45(22.5)	49(24.5)	0.89 (0.55-1.43)	0.63	0.8 (0.48 - 1.34)	0.40
GG	3(1.5)	3(1.5)	0.92 (0.18-4.73)	0.92	0.43 (0.08 - 2.46)	0.35
rs10421768 in *HAMP*						
AA	105(52.5)	125(62.5)	1	-	1	-
AG	81(40.5)	69(34.5)	1.34 (0.90-2.00)	0.15	1.33 (0.87 - 2.03)	0.18
GG	14(7)	6(3)	**2.83 (1.00-8.01)**	**0.05**	2.90 (0.97 - 8.64)	0.06
rs1050450 in *GPX1*						
CC	108(54)	100(50)	1	-	1	-
CT	80(40)	92(46)	0.79 (0.52-1.21)	0.27	0.78 (0.5 - 1.21)	0.26
TT	12(6)	8(4)	1.50 (0.54-4.21)	0.44	1.54 (0.52 - 4.59)	0.44

rs1001179 in *CAT*						
CC	110(55)	115(57.5)	1	-	1	-
CT	77(38.5)	77(38.5)	1.06 (0.69-1.63)	0.78	1.14 (0.73 - 1.78)	0.56
TT	13(6.5)	8(4)	1.68 (0.68-4.17)	0.26	1.64 (0.64 - 4.23)	0.31
rs4880 in *SOD2*						
AA	50(25)	47(23.5)	1	-	1	-
AG	95(47.5)	106(53)	0.83 (0.51-1.36)	0.47	0.82 (0.49 - 1.36)	0.43
GG	55(27.5)	47(23.5)	1.09 (0.64-1.88)	0.75	1.07 (0.61 - 1.89)	0.81

[a]OR_{uni} univariable conditional logistic regression

[b]OR_{multi} multivariable conditional logistic regression (adjusted for iron concentration).
