# Supplementary figures and images for: Iron levels, genes involved in iron metabolism and antioxidative processes and lung cancer incidence
Source: PLoS One. 2019 Jan 14;14(1):e0208610. doi: 10.1371/journal.pone.0208610 (PMC6331102; doi:10.1371/journal.pone.0208610)

S1 Fig. Kaplan-Meier curves of 8-years survival depending on iron level

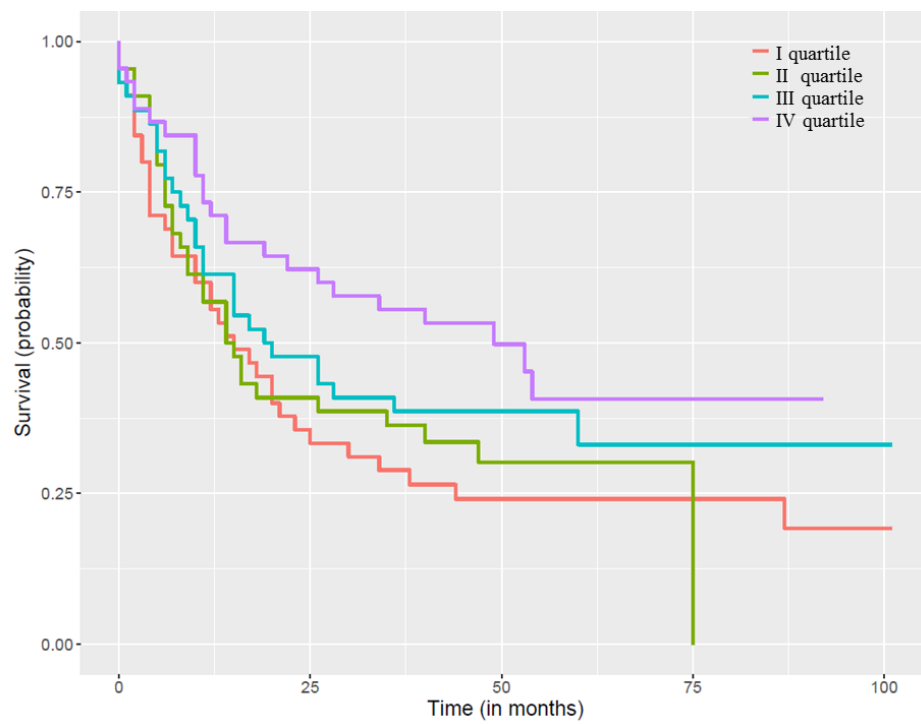

Supplement: S1 Fig — (PDF) [file pone.0208610.s008.pdf]

S2 Fig. Kaplan-Meier curves of 8-years survival depending on ferritin level

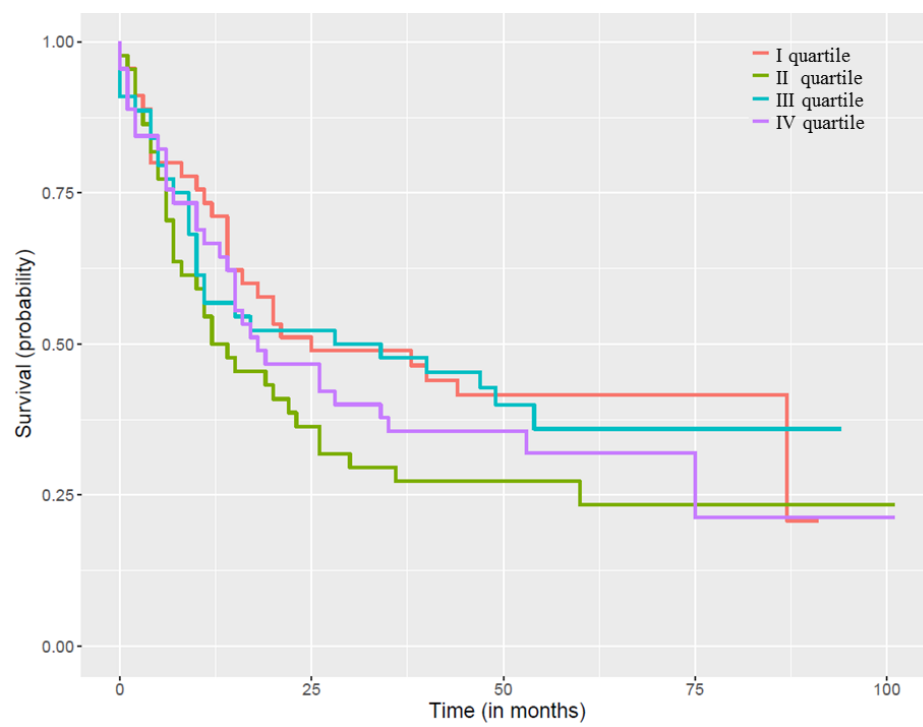

Supplement: S2 Fig — (PDF) [file pone.0208610.s009.pdf]

S3 Fig. Kaplan-Meier curves of 8-years survival depending on UIBC

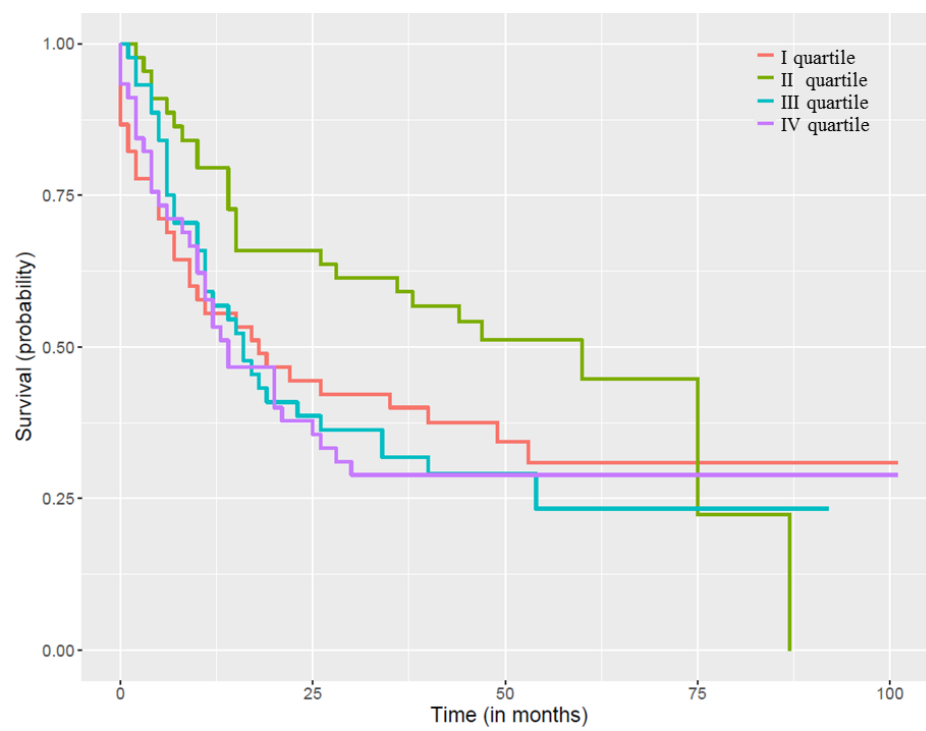

Supplement: S3 Fig — (PDF) [file pone.0208610.s010.pdf]

S4 Fig. Kaplan-Meier curves of 8-years survival depending on TIBC

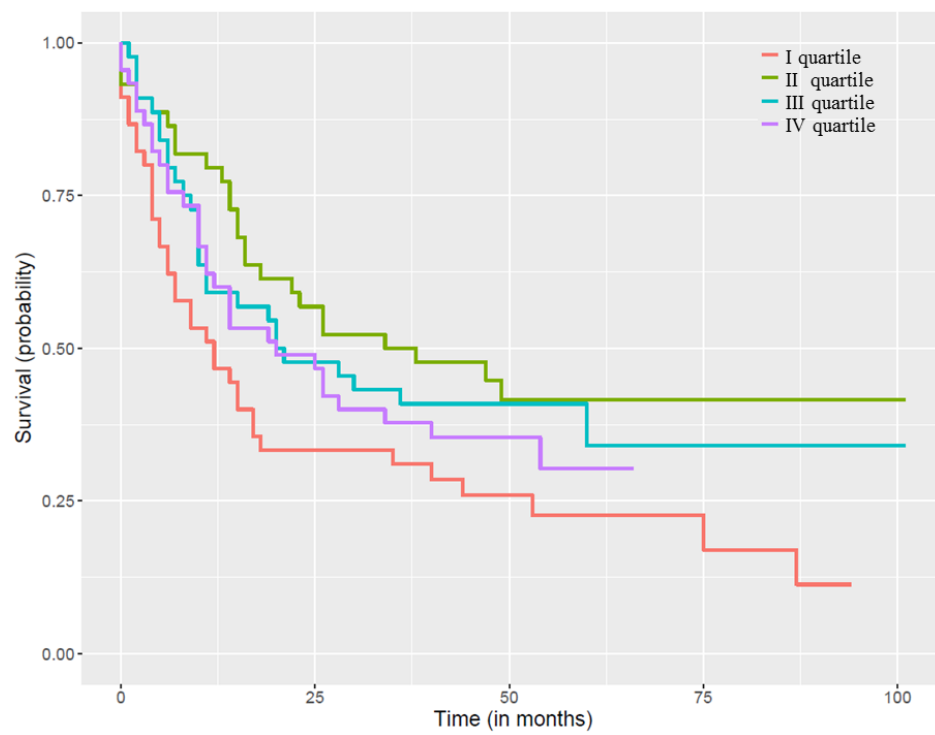

Supplement: S4 Fig — (PDF) [file pone.0208610.s011.pdf]

S5 Fig. Kaplan-Meier curves of 8-years survival depending on **transferrin saturation**

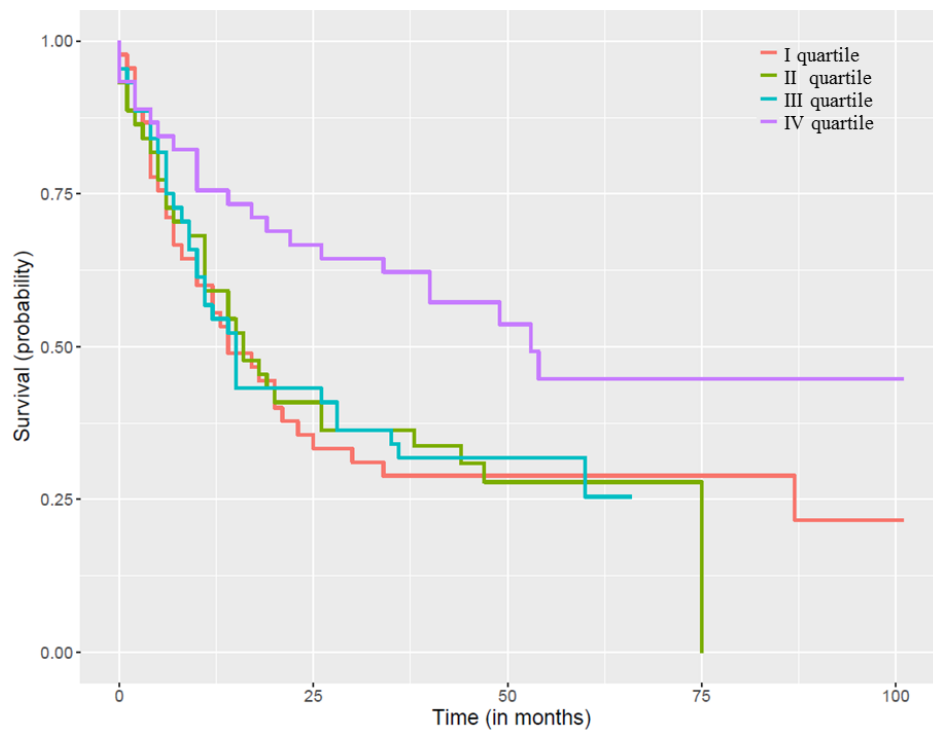

Supplement: S5 Fig — (PDF) [file pone.0208610.s012.pdf]
